# Cognition in the First Year After a Minor Stroke, Transient Ischemic Attack, or Mimic Event and the Role of Vascular Risk Factors

**DOI:** 10.3389/fneur.2020.00216

**Published:** 2020-04-21

**Authors:** Korinne Nicolas, Christopher Levi, Tiffany-Jane Evans, Patricia T. Michie, Parker Magin, Debbie Quain, Andrew Bivard, Frini Karayanidis

**Affiliations:** ^1^Functional Neuroimaging Laboratory, School of Psychology, University of Newcastle, Newcastle, NSW, Australia; ^2^Brain and Mental Program, Hunter Medical Research Institute, Newcastle, NSW, Australia; ^3^Priority Research Centre for Stroke and Brain Injury, University of Newcastle, Newcastle, NSW, Australia; ^4^University of New South Wales, Sydney, NSW, Australia; ^5^Sydney Partnership for Health, Education, Research and Enterprise, Sydney, NSW, Australia; ^6^Melbourne Brain Center, Royal Melbourne Hospital, University of Melbourne, Melbourne, VIC, Australia

**Keywords:** cognition, transient ischemic attack, minor stroke, vascular risk factors, cognitive impairment

## Abstract

**Background:** Cognitive impairment following a minor stroke or transient ischemic attack (TIA) is common; however, due to diagnostic difficulties, the prevalence and underlying cause of impairment remain poorly defined. We compared cognition in patients after a minor stroke, TIA, or mimic event at three time points in the first year following the event. We examine whether cognitive impairment occurs following these events and whether this impairment differs based on the event type. Further, we measure whether these findings persist after controlling for age, education, and the presence of vascular risk factors and whether the presence of vascular risk factors, independent of event etiology, is associated with cognitive impairment. Lastly, we investigate whether increased stroke risk, as assessed by the ABCD2, is associated with reduced cognition.

**Methods:** Medical information, a cognitive screening test, and a measure of executive functioning were collected from 613 patients (123 minor stroke, 175 TIA, and 315 mimics) using phone interviews at three time points in the first year following the event. Linear mixed models were used to determine the effect of event type, vascular risk factors, and predicted stroke risk on cognitive performance while controlling for confounders.

**Results:** There was no relationship between event type and performance on either cognitive measure. When all confounders are controlled for, performance on the cognitive screening test was uniquely accounted for by the presence of heart failure, myocardial infarction, angina, and hypertension (all *p* < 0.047), whereas the measure of executive functioning was uniquely accounted for by the presence of hypertension and angina (all *p* < 0.032). Increased stroke risk also predicted performance on the cognitive screening test and the measure of executive functioning (all *p* < 0.002).

**Conclusions:** Our findings indicate that cognitive impairment following a minor stroke or TIA may be attributed to the high prevalence of chronic vascular risk factors in these patients. This highlights the importance of long-term management of vascular risk factors beyond event recovery to reduce the risk of cognitive impairment. Increased stroke risk (i.e., ABCD2 score) was also associated with reduced cognition, suggesting that it may be helpful in signaling the need for further cognitive evaluation and intervention post-event.

## Introduction

Minor stroke and transient ischemic attack (TIA) are brief episodes of neurological dysfunction that lie on a spectrum of severity, with minor stroke considered a more severe neurological event (1). While clinical symptoms are transient, there is increasing evidence of persistent moderate cognitive impairment (2, 3). Moreover, patients frequently complain of persistent memory problems, confusion, and fatigue (4) that interfere with activities of daily living and reduce quality of life (5). The rate of moderate (29–68%) and severe (5–22%) cognitive impairment varies across studies (3). This variability is likely to be partly due to clinical factors that affect diagnosis, as symptoms are often resolved before clinical assessment and diagnosis is often based on clinician's interpretation of the patients' recollection of the event. Moreover, as some non-vascular conditions (e.g., episodes of migraine, seizure, or vestibular disturbance) mimic the symptoms of minor stroke and TIA (6), it is possible that some studies that do not include specialist diagnosis may inadvertently include a proportion of mimic patients for whom changes in cognitive functioning have not been specifically established.

Estimates of cognitive impairment in minor stroke and TIA patients may also be inflated by the failure to control for the pre-event presence of vascular (e.g., hypertension and hyperlipidemia) and non-vascular (e.g., age and education level) risk factors that are known to impact cognitive functioning (7). Vascular risk factors accelerate the progression of atherosclerosis, resulting in decreased blood flow and higher risk of white matter damage (8) and vascular events (9). White matter damage associated with vascular risk factors is most prominent in the prefrontal cortex, an area that supports executive functions (2, 10). Age-related changes in brain structure and cognition are accelerated 2-fold over a 5-year period in people with vascular risk factors compared to age-matched controls (11, 12). Minor stroke and TIA patients have a greater prevalence of vascular risk factors than do healthy controls (2, 13) and a greater rate of neural atrophy over a 3-year period (14). Therefore, cognitive complaints associated with these minor neurological events may, in fact, arise from more chronic vascular disease processes predating or following on from the focal ischemic damage caused by the event itself.

In this study, we investigate cognitive functioning during the first year after a minor stroke, a TIA, or a mimic event. We examine whether cognitive functioning varies as a function of event type and, if so, whether these differences can be attributed to the differential presence of vascular risk factors. As a minor stroke is a more severe vascular event than a TIA, we expect that it will be associated with poorer cognitive performance. Likewise, we expect that TIA will be associated with poorer cognition than the range of transient mild symptoms that characterize mimic events. We expect that vascular risk factors will also be associated with poorer cognitive ability, independently of event etiology. However, if the neurological impact of minor stroke and TIA is greater than the impact of vascular risk factors, event type effects on cognition are expected to persist when controlling for vascular risk factors. Finally, we examine the clinically important question of whether risk of future stroke, assessed using the ABCD2, can predict cognition, independently of event type and vascular risk factors.

## Methods

The International Systems of Care and Patient Outcomes in Minor Stroke and TIA (INSIST) is a longitudinal, community-based inception cohort study that was approved by the Hunter New England Health and the University of Newcastle Human Research Ethics Committees (12/04/18/4.02; H-2012-0154). All patients provided written informed consent.

Six hundred and thirteen patients who presented to 16 primary and secondary care practices, including general practices, emergency departments, acute neurovascular clinics, stroke units, and hospitals in the Hunter and Manning Valley regions of New South Wales, Australia, with a possible stroke or TIA diagnosis were recruited into the study (15).

Adjudication of the presenting event as a minor stroke, TIA, or mimic event was completed by a specialist clinical panel including two stroke neurologists, a general practitioner, and an academic neuroradiologist using a standardized clinical definition across all practices (see Table 1). Adjudication was based on a consensus decision across the panel using the patient's narrative of the baseline event, clinical symptom duration, and type (i.e., positive or negative), modality (e.g., motor or sensory), and medical information (i.e., neuroimaging, clinical, and radiological information). As the study recruited from primary care facilities without access to medical imaging facilities, clinical MRI was unavailable for many patients.

**Table 1:** Criteria for adjudication of a TIA, minor stroke, or mimic event in the INSIST study.

**Adjudicated group**	**INSIST criteria**
Transient ischemic attack	Rapidly developing clinical signs of focal disturbance of cerebral function lasting less than 24 h with no apparent non-vascular cause
Minor stroke	A stroke with a National Institutes of Health Stroke Scale score of less than or equal to 4 lasting more than 24 h
Mimic event	Neurological disturbance with no apparent vascular cause

At baseline, 123 events were adjudicated as a minor stroke, 175 as a TIA, and 315 as a mimic (see Supplementary Table 1 for information on neurovascular event subtype, territory, and mimic diagnosis). Recurrent events that occurred after the baseline event were clinically adjudicated at each interview using the same adjudication panel, criteria, and medical information as baseline events. However, as the focus of this study was to examine the effect of a baseline event on cognition, only the patient's baseline event diagnosis was used to determine event type for this study.

### Procedure

Following their index event, participants completed three phone interviews which included demographic, neurological, cognitive, and affective measures. Due to logistical factors in recruitment and interview scheduling, the time from the baseline event to each interview varied across participants. As a result, we used time since event rather than interview occasion to assess change in outcomes post-event. Specifically, time was converted into a categorical variable based on days since the baseline event, producing three time points. Time Point 1 included interviews conducted 80 days post-event, Time Point 2 included interviews conducted 81–335 days post-event, and Time Point 3 included interviews conducted more than 335 days post-event (maximum 1,000 days; see Table 2). The majority of first, second, and third interviews were included in time points 1, 2, and 3, respectively.

**Table 2:** Summary of recategorization from interview occasion (columns) to time points (rows) used to analyze time since event in lieu of interview occasions.

		**Interview occasion**			
		**1**	**2**	**3**	
Days post-event (mean ± SD)		57 ± 50	150 ± 53	381 ± 41	
**Time point**	**Days post-event**	***n***	***n***	***n***	**Total** ***N***
1	0–80	497	0	1	498
2	81–335	113	603	7	723
3	336+	3	5	603	611
Total *N*		613	608	611	1,832

#### Dependent Variables: Cognitive Functioning

The Telephone Interview for Cognitive Status [TICS; (16)] is an 11-item cognitive screening test (maximum score 41). The TICS has been validated against the Mini-Mental State Examination (17).

The semantic Verbal Fluency Task [VFT; animal names in 1 min, (18)] was used as a measure of executive functions. It has been validated for use over the telephone (19) with scores over 13 indicating healthy performance in older adults (20).

#### Independent Variables: ABCD2 Score, Vascular Risk Factors, Patient Demographics, Mood

The ABCD2 score is a risk stratification tool designed to identify the predicted risk of stroke in patients who present to acute care with a minor stroke or a TIA. The ABCD2 is used as a guide to emergency assessment and treatment, predicting the risk of stroke at 2, 7, and 90 days post-event (21). The ABCD2 combines significant clinical features of a minor neurological event and vascular risk factors to categorize a patient's risk of future stroke. As is common in the literature (22, 23), ABCD2 scores were analyzed as a categorical variable with three groups, each indicating a different risk of future stroke: low (scores of 0–3), moderate (scores of 4–5), and high (scores of 6–7).

Patient demographics included age at the time of event, sex, years of education, and the presence of vascular risk factors (hypertension, hyperlipidemia, myocardial infarction, heart failure, angina, peripheral vascular disease, atrial fibrillation, and diabetes). The presence of vascular risk factors at baseline was based on clinical diagnosis by the attending clinician.

The Hospital Anxiety and Depression Scale [HADS, (24)] is a self-assessment scale for depression and anxiety in a medical environment with good psychometric properties in psychiatric, primary care patients and the general population (25). A score of >11 (range 0–21) on the HADS indicates a higher risk of depression (24).

The modified Rankin Scale [mRS, (26)] measures the degree to which physical disability following a stroke impedes daily activities. Scores range from 0 to 6, with higher scores indicating greater levels of disability. mRS scores were obtained at each time point, and a pre-event mRS score was derived retrospectively using information from patients and carers. Pre- and post-event mRS scores show good concurrent validity and are a robust predictor of prognosis (27, 28).

### Statistics

Event type effects (minor stroke, TIA, and mimic) were examined using a one-way ANOVA for continuous variables (age), a Kruskal–Wallis *H* test for discrete variables (number of vascular risk factors, categorized ABCD2 score, and mRS), and a chi-square test of independence for categorical variables [attrition, vascular risk factor presence, sex, depression (>11 on the HADS), and low education (≤12 years of formal education), (29)] and summarized using the mean and standard deviation. Significant main effects of event type were examined using simple contrasts between group pairs. *Post-hoc* power analysis found that the INSIST sample size had 80% power to detect a moderate effect size of 0.35 (Cohen's *D*) for these continuous outcomes when comparing any two groups.

Linear mixed models were built in Stata (version 14) to examine crude main effects of each independent variable (IV; event type, grouped ABCD2 score, and vascular risk factors) on each dependent variable (DV; TICS and VFT) across time points. The crude model for each IV also included a term for time point as a categorical variable and a random effect to control for the correlation of repeated measures on individuals. Adjusted models were created to examine whether any significant main effect of the IV survived after adjusting for potential confounders for each IV–DV relationship.

Directed acyclic graphs (DAGs) for each model were derived to theoretically characterize the relationship between each IV and DV and to identify confounders that needed to be controlled in order to estimate the total effect of the IV on the DV (30). Based on the DAG, to identify the independent effect of event type on cognition (Model 1), we needed to control for age and all vascular risk factors. For each vascular risk model (Model 2), we needed to control for education, depression, age, and all other vascular risk factors. For these models, the DAG identified that event type was a potential mediator. To examine whether event type affected the relationship between vascular risk factors and cognition, in initial analyses, event type was included in each vascular risk model. As its inclusion scarcely changed findings, event type was not included in the vascular risk models presented below. For the ABCD2 model (Model 3), we controlled for event type and all vascular risk factors excluding hypertension and diabetes, as they contribute to the ABCD2 score. For all models, there was no evidence of violation of the model assumptions of linearity, non-constant variance, and normality of the residuals.

## Results

Of the 613 participants, two were missing both TICS and VFT scores and were removed from further analyses (*N* = 611). We used mixed model analysis for these longitudinal data, so data from all 611 participants contributed to the analyses, even if there were missing data on one or two time points. Table 3 summarizes the number of participants who completed the TICS and VFT at each interview occasion.

**Table 3:** Summary of the number of participants who provided cognitive measures at each interview.

**Number of interview(s) completed**	**TICS**	**VFT**
Interview 1 Only	20 (3.3%)	20 (3.3%)
Interview 2 Only	1 (0.2%)	3 (0.5%)
Interview 3 Only	0	1 (0.2%)
Interviews 1 and 2	25 (4.1%)	27 (4.4%)
Interviews 1 and 3	12 (2.0%)	15 (2.5%)
Interviews 2 and 3	1 (0.2%)	14 (2.3%)
All interviews	552 (90.3%)	531 (86.9%)
Total data obtained	611	611

Twenty participants completed the TICS and VFT only on their first interview occasion and were thereafter lost to follow-up. These 20 participants were older (*p* < 0.001) and performed more poorly on both the TICS and VFT measures at Time Point 1 (*p* = 0.001 and *p* = 0.036, respectively; data not tabulated) than participants who contributed to additional data points. Excluding these participants from all analyses below did not change the pattern of findings, so they were retained.

As shown in Table 4, the three event types differed significantly on a number of demographic and risk variables. There were significant group differences in sex (*p* < 0.001), ABCD2 categories (*p* < 0.001), number of vascular risk factors (*p* < 0.001), and prevalence of all vascular risk factors excluding hyperlipidemia and diabetes (all *p* < 0.033). Comparisons (data not tabulated) revealed that minor stroke and TIA patients had a higher percentage of males compared with mimic patients (*p* < 0.001). Minor stroke patients had significantly higher ABCD2 scores and number of vascular risk factors compared to both TIA and mimic patients, and TIA patients had higher ABCD2 scores and number of vascular risk factors compared to mimic patients (all *p* < 0.001). Group differences regarding the presence of vascular risk factors were driven by a greater prevalence in minor stroke and TIA patient groups (see Table 4).

**Table 4:** All participants' sociodemographic, vascular, and lifestyle risk factors by event type.

	**Minor stroke**	**TIA**	**Mimic**	**Total**	***F*/χ^**2**^**	***df***	***p***	**Direction of significant effect**
	***N* = 123 (20%)**	***N* = 175 (29%)**	***N* = 315 (51%)**	***N* = 611**				
Age (Mean ± SD)	73 ± 10	72 ± 11	68 ± 13	70 ± 12	1.20	2, 611	0.153	Mi < TIA^***^
								Mi < MS^***^
Sex (% Male)	67 (54%)	104 (59%)	107 (34%)	278 (45%)	34.6	2	<0.001	Mi < TIA^***^
								Mi < MS^***^
**Grouped ABCD2 (% Yes)**								
Low risk (0–3)	17 (14%)	92 (53%)	202 (64%)	311 (51%)	91.0	2	<0.001	Mi < TIA^***^
Moderate risk (4–5)	65 (53%)	59 (34%)	95 (30%)	219 (36%)				Mi < MS^***^
High risk (6–7)	41 (33%)	23 (13%)	14 (4%)	78 (25%)				TIA < MS^***^
**Vascular risk factor (% Yes)**								
Hypertension	100 (81%)	123 (70%)	202 (64%)	425 (70%)	12.4	2	0.002	Mi < MS^***^
Hyperlipidemia	70 (57%)	96 (55%)	155 (49%)	321 (52%)	2.7	2	0.257	Mi < MS^***^
Myocardial infarction	27 (22%)	22 (13%)	24 (8%)	73 (12%)	17.4	2	<0.001	TIA < MS^*^
Heart failure	16 (13%)	17 (10%)	18 (6%)	51 (8%)	6.8	2	0.033	Mi < MS^*^
Angina	30 (25%)	31 (18%)	40 (13%)	101 (17%)	9.1	2	0.011	Mi < MS^*^
Peripheral vascular disease	11 (9%)	18 (10%)	11 (3%)	40 (7%)	10.0	2	0.007	Mi < TIA^**^
Atrial fibrillation	26 (21%)	41 (23%)	35 (11%)	102 (17%)	14.6	2	0.001	Mi < TIA^**^
Diabetes	29 (24%)	30 (17%)	47 (15%)	106 (17%)	4.6	2	0.098	Mi < MS^*^
No. vascular risk factors (mean ± SD)	2.51 ± 1.64	2.16 ± 1.52	1.69 ± 1.35	1.99 ± 1.49	27.5	2	<0.001	Mi < TIA^***^
								Mi < MS^***^
								TIA < MS^***^
**Lifestyle risk factor (% Yes)**								
Low education	59 (48%)	86 (49%)	135 (43%)	280 (46%)	2.1	2	0.347	
Depression	13 (11%)	17 (10%)	27 (9%)	57 (9%)	1.4	2	0.492	

* p < 0.05,

** p < 0.010,

*** p < 0.001,

*Mi, mimic; MS, minor stroke. Shaded p-values indicate p < 0.05*.

### Cognitive and Disability Changes Across Time Points

As shown in Table 5, TICS scores increased progressively over post-event time points [*N* = 611, χ^2^(2) = 74.17, *p* < 0.001; Time Points 1 to 2: 95% CI, −1.11 to −0.28; Time Points 2 to 3: 95% CI, 0.02 to 0.83]. Although statistically significant, the total increase of 1.1 score (maximum = 41) is not clinically significant. VFT scores did not vary across time points [*N* = 611, χ^2^(2) = 0.65, *p* = 0.724]. mRS scores varied significantly over time [*N* = 611, χ^2^(3) = 92.67, *p* < 0.001]. There was a substantial increase in disability from pre-event to Time Point 1 (95% CI, 0.11 to 0.21) and a smaller but progressive increase thereafter (Time Points 1 to 2: 95% CI, 0.12 to 0.22; Time Points 2 to 3: 95% CI, 0.18 to 0.28). Depression scores varied significantly over time [*N* = 611, χ^2^(2) = 32.71, *p* < 0.001], decreasing slightly from Time Points 1 to 2 (95% CI, −0.49 to 0.17) and significantly from Time Points 2 to 3 (95% CI, −0.94 to −0.25).

**Table 5:** Mean cognitive and disability measures over time points.

	**Minor stroke**	**TIA**	**Mimic**	**χ^**2**^**	***df***	***p***
**Telephone interview for cognitive status (Mean ± SD)**						
Time 1	32.85 ± 3.83	33.47 ± 3.83	33.82 ± 3.53	74.2	2	<0.001
Time 2	33.76 ± 3.65	34.53 ± 3.48	34.21 ± 3.45			
Time 3	34.59 ± 4.01	34.53 ± 3.56	34.72 ± 3.74			
Mean over time	33.73 ± 3.83	34.18 ± 3.62	34.25 ± 3.57			
**Verbal fluency test (Mean ± SD)**						
Time 1	17.65 ± 5.65	18.78 ± 5.62	20.23 ± 6.15	0.6	2	0.724
Time 2	18.86 ± 6.45	19.27 ± 6.45	19.60 ± 6.28			
Time 3	18.75 ± 6.39	18.72 ± 6.42	19.88 ± 6.40			
Mean over time	18.42 ± 6.16	18.92 ± 6.16	19.90 ± 6.28			
**Modified rankin scale (Mean ± SD)**						
Pre-event	0.72 ± 1.13	0.75 ± 1.08	0.68 ± 1.02	92.7	2	<0.001
Time 1	1.25 ± 1.11	0.76 ± 1.05	0.76 ± 1.04			
Time 2	1.27 ± 1.16	0.82 ± 1.12	0.76 ± 1.03			
Time 3	1.37 ± 1.15	0.93 ± 1.16	0.78 ± 1.07			
Mean over time	1.15 ± 1.14	0.82 ± 1.10	0.75 ± 1.04			
**Hospital anxiety and depression scale (Depression component) (Mean ± SD)**						
Time 1	3.31 ± 0.19	2.91 ± 0.17	2.94 ± 0.15	32.7	2	<0.001
Time 2	3.16 ± 0.18	2.75 ± 0.15	2.78 ± 0.13			
Time 3	2.72 ± 0.18	2.31 ± 0.17	2.34 ± 0.14			
Mean over time	3.06 ± 0.18	2.66 ± 0.16	2.69 ± 0.14			

*Shaded p-values indicate p < 0.05*.

Time point was included in all three models below but did not interact with either TICS or VFT in any of the three models and will not be discussed further (all *p* > 0.150; data not tabulated).

### Model 1: Event Type on Cognition

In the crude main effects model for event type (Table 6), TICS scores did not vary with event type. There was a small but significant effect of event type on VFT, with simple comparisons indicating that minor stroke patients performed more poorly than mimic patients (*p* = 0.008; data not tabulated). Including age as a potential confounder (adjusted model) reduced all coefficients and removed the relationship between VFT scores and event type. The final TICS and VFT models that controlled for both age and vascular risk factors resulted in further reduced coefficients. The addition of depression in the model scarcely affected coefficients from those presented and was not included in any of the event type models.

**Table 6:** Effects of event type on TICS and VFT.

**Cognitive measures by event type**									
	**Crude**			**Adjusted**			**Final**		
**Outcome**	***β***	**95% CI**	***p***	***β***	**95% CI**	***p***	***β***	**95% CI**	***p***
**Telephone interview for cognitive status**									
MS–TIA	−0.60	−1.4 to 0.2	0.213	−0.56	−1.3 to 0.2	0.252	−0.32	−1.2 to 0.1	0.191
Mimic–TIA	0.00	−0.6 to 0.6		−0.41	−1.0 to 0.2		−0.53	−2.3 to 0.7	
MS–Mimic	−0.60	−1.2 to 0.1		−0.15	−0.8 to 0.5		0.21	-0.4 to 0.8	
**Verbal fluency test**									
MS–TIA	−0.69	−2.0 to 0.6	0.022	−0.60	−1.8 to 0.6	0.441	−0.24	−1.4 to 0.9	0.910
Mimic–TIA	0.87	-0.1 to 1.9		0.10	−0.9 to 1.1		−0.04	−1.0 to 0.9	
MS–Mimic	−1.60	−2.7 to −0.4		−0.70	−1.8 to 0.4		−0.20	−1.3 to 0.9	

*Left: Crude models including event type and time point. Middle: Adjusted model including time and controlling for age. Right: Final model including time and controlling for age and vascular risk factors. Shaded beta values indicate significant confidence intervals and significant findings (α < 0.05)*.

### Model 2: Vascular Risk Factors on Cognition

Table 7 shows that both TICS and VFT scores were significantly lower in the presence of most individual vascular risk factors. For TICS scores, the strongest predictor was heart failure, followed in decreasing order by myocardial infarction, angina, peripheral vascular disease, hypertension, and atrial fibrillation. For VFT, the strongest predictor was again heart failure, followed by hypertension, angina, myocardial infarction, peripheral vascular disease, atrial fibrillation, and hyperlipidemia. Controlling for age and education reduced the strength of the effect of each vascular risk factor on both TICS and VFT. The final models for each vascular risk factor also controlled for all other vascular risk factors. As shown in Table 7, when controlling for other vascular risk factors, the only vascular risk factors to uniquely significantly predict TICS were, in descending order, heart failure, myocardial infarction, angina, and hypertension. For VFT scores, only hypertension and angina remained as unique predictors. The addition of depression and event type scarcely altered coefficients from those presented above and therefore were not included in any of the vascular risk factor models.

**Table 7:** Effects of vascular risk factors on TICS and VFT.

**Cognitive measures by vascular risk factors**									
	**Crude**			**Adjusted**			**Final**		
**Outcome**	***β***	**95% CI**	***p***	***β***	**95% CI**	***p***	***β***	**95% CI**	***p***
**Telephone interview for cognitive status**									
Hypertension	−1.43	−2.0 to −0.9	<0.001	−0.74	−1.3 to −0.2	0.009	−0.68	−1.3 to −0.1	0.020
Hyperlipidemia	−0.36	−0.9 to 0.2	0.183	−0.12	−0.6 to 0.4	0.633	0.28	−0.2 to 0.8	0.296
Myocardial infarction	−1.93	−2.7 to −1.1	<0.001	−1.36	−2.1 to −0.6	<0.001	−0.88	−1.7 to −0.1	0.004
Heart failure	−2.48	−3.4 to −1.5	<0.001	−1.5	−2.4 to −0.6	0.001	−0.96	−1.9 to 0.0	0.047
Angina	−1.84	−2.5 to −1.1	<0.001	−1.21	−1.9 to −0.5	<0.001	−0.77	−1.5 to 0.0	0.038
Peripheral vascular disease	−1.77	−2.8 to −0.7	0.001	−0.8	−1.8 to 0.2	0.119	0.01	−1.0 to 1.1	0.983
Atrial fibrillation	−1.13	−1.8 to −0.4	0.002	−0.3	−1.0 to 0.4	0.394	−0.08	−0.8 to 0.6	0.804
Diabetes	−0.42	−1–1 to 0.3	0.245	−0.33	−1.0 to 0.3	0.307	−0.08	−0.7 to 0.6	0.804
**Verbal fluency test**									
Hypertension	−3.01	−3.9 to −2.1	<0.001	−1.69	−2.6 to −0.8	<0.001	−1.39	−2.3 to −0.5	0.004
Hyperlipidemia	−1.52	−2.4 to −0.6	<0.001	−0.98	−1.8 to −0.2	0.017	−0.39	−1.2 to 0.5	0.368
Myocardial infarction	−2.5	−3.9 to −1.1	<0.001	−1.43	−2.7 to −0.2	0.024	−0.53	−1.9 to 0.8	0.436
Heart failure	−3.98	−5.6 to −2.4	<0.001	−2.25	−3.7 to −0.8	0.003	−1.46	−3.0 to 0.1	0.067
Angina	−2.90	−4.1 to −1.7	<0.001	−1.77	−2.9 to −0.7	0.001	−1.31	−2.5 to −0.1	0.032
Peripheral vascular disease	−2.21	−4.0 to −0.4	0.016	−0.55	−2.2 to 1.1	0.516	0.74	−1.1 to 2.5	0.404
Atrial fibrillation	−2.04	−3.2 to −0.9	<0.001	−0.53	−1.6 to 0.6	0.348	−0.34	−1.4 to 0.8	0.555
Diabetes	−0.86	−2.0 to 0.3	0.146	−0.33	−1.7 to 0.4	0.219	−0.14	−1.2 to 0.9	0.798

*Left: Crude models for each vascular risk factor including only time. Middle: Adjusted model including time and controlling for age and education. Right: Final model including time and controlling for age, education, and all other vascular risk factors. Shaded beta values indicate significant confidence intervals and significant findings (α < 0.05)*.

### Model 3: ABCD2 on Cognition

Table 8 shows that greater risk of stroke was associated with significantly lower TICS and VFT scores. All relationships remained significant when controlling solely for event type (adjusted model) and together with vascular risk factors (final model). Simple comparisons (data not tabulated) revealed significant differences on TICS scores between all groups (low risk vs. moderate risk *p* = 0.008, low vs. high *p* < 0.001, and moderate vs. high *p* = 0.036) and on VFT scores between low- and moderate-risk groups (*p* = 0.002) and low- and high-risk groups (*p* = 0.009). Performance on the VFT did not differ between moderate- and high-risk groups (*p* = 0.572). The addition of depression in the model scarcely affected coefficients from those presented and was not included in any of the ABCD2 models.

**Table 8:** Effects of ABCD2 on cognition.

**Cognitive Measures by ABCD2**									
	**Crude**			**Adjusted**			**Final**		
**Outcome**	***β***	**95% CI**	***p***	***β***	**95% CI**	***p***	***β***	**95% CI**	***p***
**Telephone interview for cognitive status**									
Mod–Low	−0.82	−1.4 to −0.3	<0.001	−0.85	−1.5 to −0.2	<0.001	−0.79	−1.37 to −0.2	<0.001
High–Low	−1.77	−2.5 to −0.9		−1.82	−2.7 to −0.9		−1.7	−2.57 to −0.8	
High–Mod	−0.94	−1.8 to −0.8		−0.97	−1.9 to −0.1		−0.91	−1.8 to −0.1	
**Verbal fluency test**									
Mod–Low	−1.78	−2.7 to −0.8	<0.001	−1.6	−2.7 to −0.7	<0.001	−1.53	−2.5 to -0.6	0.002
High–Low	−2.58	−3.9 to −1.2		−2.3	−3.8 to −0.8		−1.94	−3.4 to −0.5	
High–Mod	−0.80	−2.2 to 0.6		−0.66	−2.1 to 0.8		−0.41	−1.8 to 1.0	

*Left: Crude models including ABCD2 and time point. Middle: Adjusted model including time and controlling for event type. Right: Final model including time and controlling for hyperlipidemia, myocardial infarction, heart failure, angina, peripheral vascular disease, atrial fibrillation, and event type. Shaded beta values indicate significant confidence intervals and significant findings (α < 0.05)*.

## Discussion

The present study used a cognitive screening test (TICS) and a brief measure of executive functioning (VFT) to investigate differences in the level and progression of cognitive ability in the first year after a minor stroke, TIA, or mimic event. We sought to identify whether cognitive ability was differentially impacted by event severity, independently of the presence of coexisting, chronic vascular disease processes associated with vascular risk factors.

Across the first year following a minor neurological event, there was a small but statistically significant improvement in performance on the cognitive screening test but not the measure of executive functioning. The increase in TICS score was not clinically significant; did not differ by event type, presence of vascular risk factor, or risk of future stroke; and remained when removing the few participants (3%) who only completed the first interview. This small increase in TICS may reflect slight recovery of cognition following a brief disruption after an event and/or test practice effect.

### Event Type

Event type had a significant effect on the measure of executive functioning that was driven by poorer performance in the minor stroke group compared to the mimic group. This effect was weak and was removed after controlling for either vascular risk factors or age. In conclusion, in this large sample and using broad cognitive screening tasks, differences in cognitive functioning between minor stroke, TIA, and mimic events in the first year post-event were weak and attributable to differential prevalence of vascular risk factors and/or age. Alternatively, it is possible that the broad cognitive measures used in this study were not sufficiently sensitive to detect subtle differences as a function of event type. It is possible that more sensitive cognitive tasks and functional brain imaging measures may reveal more subtle impairments.

Other studies suggest that both neural structure and cognitive function are affected in the subacute stage following a minor stroke or TIA. Bivard et al. (31) found that, compared to healthy controls with a similar number of vascular risk factors, both minor stroke and TIA patients who had evidence of acute ischemia in brain perfusion imaging showed cerebral gray matter atrophy from baseline to 90 days post-event as well as poorer performance on executive function and attention subscales of the Montreal Cognitive Assessment (MoCA). There is less consistent evidence for long-term cognitive impairment following a TIA or minor stroke. Munir et al. (14) reported that, although minor stroke and TIA patients had a higher rate of neural atrophy and slower processing speed compared to healthy controls over a 3-year period, neither memory nor executive functions were affected. Similarly, Sachdev et al. (32) found that, at 3 years post-event, minor stroke and TIA patients showed reduced verbal memory but no difference in any other cognitive domain, when compared to controls. Taken together, these findings suggest that although minor stroke and TIA may be associated with cognitive decline in the early post-event periods [e.g., 90 days in Bivard et al. (31)], the effects on cognition are only transient.

### Vascular Risk Factors

Consistent with many previous studies (33–37), the presence of vascular risk factors was associated with reduced performance on both the cognitive screening task and the executive functioning measure. When we examined the unique contribution of each vascular risk factor and controlled for age and education, performance on the cognitive screening test was significantly impaired, in decreasing order of impact, by the presence of heart failure, myocardial infarction, angina, and hypertension, whereas executive function scores were significantly impacted by hypertension and angina.

Minor stroke patients had a greater prevalence of each of these vascular risk factors that independently affected cognitive performance; however, inclusion of event type as a confounder did not impact the relationship between vascular risk factors and cognition. While it is well established that vascular risk factors affect cognition, this finding provides evidence that the reported cognitive dysfunction after a minor stroke or TIA may be attributed to the increased prevalence of vascular risk factors in such patients. Moreover, the robust findings between vascular risk factors and cognitive ability after controlling for a number of confounders suggest that these broad cognitive measures are sufficiently sensitive to progressive effects arising from vascular factors. This suggests that the null finding of event type (discussed above) is unlikely to be due to test insensitivity.

### Predicted Stroke Risk

As expected, ABCD2 scores were highest in minor stroke patients, followed by TIA and mimic patients. This finding supports the use of the ABCD2 score as a tool to assist in diagnostic decisions, especially in clinical settings that may not have access to imaging or specialist resources. Further, we found that increased predicted risk of future stroke was associated with reduced performance on both cognitive tests, even after controlling for event type and vascular risk factors (excluding hypertension and diabetes which contribute to the ABCD2 score). As there was no evidence of a relationship between cognition and either event type or diabetes, it is likely that the relationship between cognition and stroke risk is driven by the other variables that contribute to the ABCD2 score, such as age and hypertension.

## Conclusion

A large community-dwelling cohort of patients with a clinical diagnosis of minor stroke, TIA, or mimic event was assessed over the first year post-event. Level of physical disability and risk of future stroke progressively increased with event severity. However, event type was not associated with differential level of functioning on a cognitive screening test and produced only a small effect on a measure of executive functioning that was eliminated after controlling for vascular risk factors and age. In contrast, the presence of vascular risk factors and most consistently hypertension and angina, as well as predicted stroke risk, were associated with reduced performance on both the cognitive screening and the executive function measures, irrespective of event etiology and other confounders.

The absence of an effect of neurological event type on cognition, in the presence of significant effects of future stroke risk and vascular risk factors, suggests that vascular risk factors may account, at least partially, for reported levels of cognitive decline after a minor stroke or TIA. This work supports the notion that these mild neurological events have only transient effects on cognitive functioning, suggesting that the common cognitive complaints reported following such transient events may arise from the increased prevalence of chronic vascular risk factors that are present in these patients before the neurological event. This highlights the need to monitor cognitive functioning associated with vascular risk factors and, in the absence of pre-event measures, obtain estimates of pre-event cognition at the time of the presenting event (38). Finally, the ABCD2, which also loads heavily on vascular risk factors, varied across neurological event type and was associated with cognitive decline, independently of event type. This is consistent with the role of vascular risk factors in cognitive decline. Together, these findings suggest that the presence of vascular risk factors could signal the need to monitor patients' cognitive functioning trajectory and consider appropriate interventions which may aid in reducing the risk of cognitive decline by improving cognitive ability. There is emerging evidence suggesting that cognitive ability can be improved by treating vascular risk factors; for example, a review by Elias et al. (39) reports multiple studies displaying an improvement in cognitive ability after treatment with candesartan in hypertensive patients. Moreover, a review by Hajduk et al. [(40), see also (41)] reports that patients with heart failure undergoing interventions to increase cardiac function displayed cognitive improvements.

A particular strength of this study is the inclusion of a large community-dwelling cohort that consisted of patients managed entirely across a range of primary care settings as well as patients in secondary care. Event adjudication was completed by a specialist panel using general practitioner, hospital, emergency department, and specialist notes to accurately diagnose vascular risk factors. Cognitive ability was assessed using trajectory analysis at three time points after the event. Based on these findings, we conclude that clinically adjudicated mild neurological events do not significantly impact cognition in the first year post-event, whereas the presence of vascular risk factors and future stroke risk are associated with lower cognition but not differential decline over a 1-year period.

This conclusion needs to be considered in light of potential caveats that may affect its generalization. Firstly, we compared minor stroke and TIA groups against a mimic group instead of a healthy control group. While mimic patients constitute a good clinical control group, these patients had a broad range of clinical presentations and are likely to perform more poorly than a healthy control group. Secondly, in the absence of clinical imaging, it was not possible to use imaging-based categorization of minor stroke and TIA or to examine whether the location of an ischemic event differentially affected cognitive profile. Thirdly, we did not control for differential effects of clinical treatment or pre-/post-event medication history. Fourthly, we did not complete a comprehensive and in-depth neuropsychological assessment, and therefore, subtle cognitive differences may have been missed. Finally, in the absence of a pre-event measure of cognitive ability, it was not possible to differentiate between premorbid cognitive level and event-related cognitive effects.

Overall, these findings are consistent with other studies showing little evidence of persistent cognitive decline post-TIA or minor stroke (14, 32). Future work is needed to examine whether behavioral and neuroimaging measures of executive function processes and associated brain networks are more sensitive to emerging and potentially reversible levels of cognitive decline associated with these minor neurological events.

## Data Availability Statement

The datasets generated for this study are available on request to the corresponding author.

## Ethics Statement

The studies involving human participants were reviewed and approved by Hunter New England Health and the University of Newcastle Human Research Ethics Committees (12/04/18/4.02; H-2012-0154). The patients/participants provided their written informed consent to participate in this study.

## Author Contributions

KN coauthored the manuscript and drafted tables and figures. CL and PM conceived the design of the study and edited drafts. T-JE supervised data analysis. PTM and AB contributed to revisions of drafts. DQ was involved in data acquisition and management. FK is the lead author, supervisor of KN's thesis and cognitive analyses, and coauthored the manuscript.

## Conflict of Interest

The authors declare that the research was conducted in the absence of any commercial or financial relationships that could be construed as a potential conflict of interest.
