# Supplementary material for: Cognition in the First Year After a Minor Stroke, Transient Ischemic Attack, or Mimic Event and the Role of Vascular Risk Factors
Source: Front Neurol. 2020 Apr 21;11:216. doi: 10.3389/fneur.2020.00216 (PMC7186464; doi:10.3389/fneur.2020.00216)
Supplement: Supplementary file 1 [file Table_1.pdf]

Supplementary Material

Supplementary Table 1 TIA and minor stroke subtype/territory and mimic event diagnosis

**Stroke Subtype (Yes, %)**	**Minor Stroke**	**TIA**
Large Artery Atherosclerosis	7 (6%)	16 (9%)
Cardio Embolism	27 (22%)	31 (18%)
Small Vessel Occlusion	18 (15%)	9 (5%)
Stroke- other etiology	1 (1%)	3 (2%)
Stroke- undetermined etiology	64 (52%)	113 (65%)
Stroke- unable to be determined	1 (1%)	3 (2%)
Hemorrhagic Stroke	5 (4%)	0
**Event Territory**		
Anterior	69 (56%)	107 (61%)
Posterior	53 (43%)	65 (37%)
Undetermined	1 (1%)	3 (2%)
**Event Sub-Territory**		
Cortical	41 (46%)	71 (52%)
Subcortical	21 (23%)	13 (9%)
Retinal	1 (1%)	18 (13%)
Uncertain	24 (30%)	35 (26%)
**Mimic Diagnosis**	**Mimic**	
Migraine	84 (27%)	
Vestibular	39 (12%)	
Seizure	17 (5%)	
Syncope/Presyncope	47 (15%)	
Transient Global Amnesia	20 (6%)	
Encephalopathy	1 (.3%)	
Metabolic/Toxic	4 (1%)	

Visual Disturbance	9 (3%)
Isolated Cranial Nerve	7 (2%)
Non-Organic	4 (1%)
Other	24 (8%)
Uncertain	41 (13%)
Cardiac	2 (1%)
Radiculopathy	4 (1%)
Headache non-migraine	3 (1%)
Disequilibrium uncertain cause	5 (2%)
Anxiety	3 (1%)
Mononeuropathy	1 (.3%)
